# Seed Metabolism and Pathogen Resistance Enhancement in *Pisum sativum* During Colonization of Arbuscular Mycorrhizal Fungi: An Integrative Metabolomics-Proteomics Approach

**DOI:** 10.3389/fpls.2020.00872

**Published:** 2020-06-12

**Authors:** Nima Ranjbar Sistani, Getinet Desalegn, Hans-Peter Kaul, Stefanie Wienkoop

**Affiliations:** ^1^Department of Ecogenomics and Systems Biology, Faculty of Life Sciences, University of Vienna, Vienna, Austria; ^2^Department of Crop Sciences, University of Natural Resources and Life Sciences, Vienna, Austria

**Keywords:** seed metabolism, *Pisum sativum*, mycorrhizal colonization, secondary metabolites, plant stress

## Abstract

Pulses are one of the most important categories of food plants, and Pea (*Pisum sativum* L.) as a member of pulses is considered a key crop for food and feed and sustainable agriculture. Integrative multi-omics and microsymbiont impact studies on the plant's immune system are important steps toward more productive and tolerant food plants and thus will help to find solutions against food poverty. *Didymella pinodes* is a main fungal pathogen of pea plants. Arbuscular mycorrhizal fungi (AMF) promote plant growth and alleviate various stresses. However, it remained unclear as to how the AMF effect on seed metabolism and how this influences resistance against the pathogen. This study assesses the AMF impacts on yield components and seed quality upon *D. pinodes* infection on two different *P. sativum* cultivars, susceptible versus tolerant, grown in pots through phenotypic and seed molecular analyses. We found that AMF symbiosis affects the majority of all tested yield components as well as a reduction of disease severity in both cultivars. Seeds of mycorrhizal pea plants showed strong responses of secondary metabolites with nutritional, medicinal, and pharmaceutical attributes, also involved in pathogen response. This is further supported by proteomic data, functionally determining those primary and secondary metabolic pathways, involved in pathogen response and induced upon AMF-colonization. The data also revealed cultivar specific effects of AMF symbiosis that increase understanding of genotype related differences. Additionally, a suite of proteins and secondary metabolites are presented, induced in seeds of *P. sativum* upon AMF-colonization and pathogen attack, and possibly involved in induced systemic resistance against *D. pinodes*, useful for modern breeding strategies implementing microsymbionts toward increased pathogen resistance.

## Introduction

A sustainable supply of crops to feed human societies could be the most important humanitarian action and its impact on declining of current and future food restrictions on this planet should be more sensed especially in regions with the highest food insecurity. Hence, the stable food supply is completely dependent on the sustainable production of crops by increasing yield and quality improvement through the development of natural potentials locally. To supply the food sustainably, large-scale production of pulses or legumes is inevitable (Fernandez-Aparicio et al., 2010). Pea (*Pisum sativum* L.) is one of the major legumes in the world (FAOSTAT^1^, updated 2019). Moreover, the use of microsymboints is in line with sustainable agriculture (Sayeed Akhtar et al., 2011).

Considering the importance of pulses as a source of vegetable protein in food basket and global need to improve their quantity and quality sustainably should be the most urgent need particularly in regions where people are suffering from the highest poverty rate. Thus, the study of microsymbionts which affect yield components of legumes especially on seed and upon stress conditions not only due to better use of natural growth promoters in agricultural systems but also most probably reduces the losses caused by abiotic and biotic stresses. Arbuscular mycorrhizal fungi (AMF) have interactions with a broad range of plants in nature (Smith and Read, 2008, as cited in Wehner et al., 2010). AMF inoculation of crops due to an increase in yield and growth (Hamel and Plenchette, 2007, as cited in Horii and Ishii, 2014). Promoting tolerance against water deficit and growth has been observed in papaya plants associated with AMF (Cruz et al., 2000, as cited in Horii and Ishii, 2014).

Chemical properties, physiology, and seed development of soybean could be influenced by AMF root colonization (Bethlenfalvay et al., 1997). Bona et al. (2016) noted to positive effects of AMF root colonization in prior studies on quality and yield in strawberry (Castellanos-Morales et al., 2010; Castellanos-Morales et al., 2012; Lingua et al., 2013; Bona et al., 2015) and saffron (Aimo et al., 2010). Also, the inoculation of *Allium sativum* with AMF has enhanced the yield and growth (Borde et al., 2009, as cited in Bona et al., 2016).

The formed symbiosis between AMF and maize (*Zea mays*) affected its seed proteome through modulation of enzymes related to functional categories such as stress, nucleotide metabolism, energy, storage, and development (Bona et al., 2016). Colonization of *Amorpha fruticosa* roots by *Glomus mosseae* has been modified the root proteome (Song et al., 2015). The *G. mosseae* by promoting cell integrity and osmotic stress depletion in wheat protected the root system against drought stress (Bernardo et al., 2017). The protein synthesis, metal handling, RNA metabolism, and reactive oxygen species (ROS) are influenced in leaf proteome of pea plants inoculated with AMF (Desalegn et al., 2016). In leaves of AMF colonized *Medicago truncatula* plants, not only genes related to synthesis of jasmonic acid (JA), flavonoid, abscisic acid (ABA), and terpenoids were up-regulated but also flavonoids and anthocyanins were intensified (Adolfsson et al., 2017).

Biotic stresses such as plant diseases are serious threats for food plants and consequently for food sources (Strange and Scott, 2005). One of the main diseases known in *P. sativum* is ascochyta blight caused by *D. pinodes* (Moussart et al., 1998). *D. pinodes* as the most abundant pathogen among seeds of *P. sativum* (Deneufbourg et al., 1994, as cited in Moussart et al., 1998) causes the poor quality and yield reduction (Khan et al., 2013). Under the field conditions, disease management of this pathogen is difficult in comparison with the control of other diseases upon *P. sativum* (Khan et al., 2013).

Chemical treatment of crop seeds is considered one of the major strategies in integrated control of ascochyta blight (Davidson and Kimber, 2007). On the other hand, using chemical compounds is not only a sustainable solution for the control of plant diseases but also acts as a destroyer of the environment or against sustainable agriculture systems. Therefore, symbiosis formation between food plants and microsymbionts with bio-control potential could be used as stable and safe disease management. Promoting of resistance against biotic stress in plants by AMF is proved (Azcón-Aguilar and Barea, 1996, as cited in Rebollo Couto et al., 2013).

Dehne (1982) reviewed the former studies on the role of mycorrhizae for effective protection of varied plant species against fungal pathogens such as *Pythium ultimum* (Stewart and Pfleger, 1977)*, Phytophthora cinnamomi* (Bäertschi et al., 1982), *P. megasperma* (Chou and Schmitthenner, 1974), *P. parasitica* (Schenck et al., 1977; Davis and Menge, 1980), *F. oxysporum cucumerinum* (Dehne, 1977), *F. oxysporum lycopersici* (Dehne and Schoenbeck, 1979), *Rhizoctonia solani* (Stewart and Pfleger, 1977), *Cylindrocladium scoparium* (Barnard, 1977), *Phoma terrestris* (Becker, 1976), *Pyrenochaeta terrestris* (Safir, 1968), *Olpidium brassicae* (Schoenbeck and Dehne, 1979; Schoenbeck and Dehne, 1981), and *Thielaviopsis basicola* (Baltruschat and Schonbeck, 1972; Schoenbeck and Dehne, 1977).

Replacing common chemical fertilizers and toxicant with AMF has been focused in line with sustainable agriculture (Gange et al., 1999; Harrier and Watson, 2004; Wang et al., 2008; Lecomte et al., 2011, as cited in Rebollo Couto et al., 2013). Additionally, AMF could be applied as a bio-control agent and growth promoter (Sayeed Akhtar et al., 2011). It was found that AMF, directly and indirectly, inhibit the prevalence of fungal root pathogens (Wehner et al., 2010). The role of AMF in strengthening the host plant upon pathogen infection by up-regulation of defensive pathways and signals has been described (Haneef Khan et al., 2010).

Some studies have investigated the AMF impacts on the plant's growth at the maturity stage (Bethlenfalvay et al., 1994; Bethlenfalvay et al., 1997; Al-Karaki and Clark, 1999). Prior researches have mainly investigated the role of AMF on soil-borne pathogens in non-legumes and few investigations have attended to AMF impacts as a bio-control agent on aboveground fungal pathogens including ascochyta blight of *P. sativum* caused by *D. pinodes*. Further, most studies have analyzed the growth parameters of host plants upon mycorrhizal colonization rather than yield components. For instance, recent studies that have investigated the *D. pinodes* infection of *P. sativum* upon different treatments including mycorrhizal plants, were focusing on the leaves where no clear disease reduction in mycorrhizal treatments compared with non-mycorrhizal was reported (Desalegn et al., 2016; Turetschek et al., 2017). In general, only a few studies exist, using multilevel-omics approaches by integrating plant morphological and molecular phenotyping (Zivy et al., 2015).

Here, we assess the integrative effects of AMF on several yield components, growth parameters, seed secondary metabolome, and -proteome upon tolerant and susceptible pea cultivars against *D. pinodes* as an aboveground pathogen in pots. This study answers the following research questions: I) Does AMF affect above-belowground growth and yield parameters in pea plants? II) How does AMF promote the productivity and seed quality of *P. sativum* from the phenotypic-omics perspective? III) Has AMF-symbiosis a protecting effect on seed quality of tolerant and susceptible pea cultivars upon pathogen attack? IV) Do AMF influence the seed secondary metabolome and proteome? and V) How do AMF affect the response of the seed metabolism upon pathogen infection?

## Materials and Methods

### Performing Experiments and Planting

**Figure 1:**
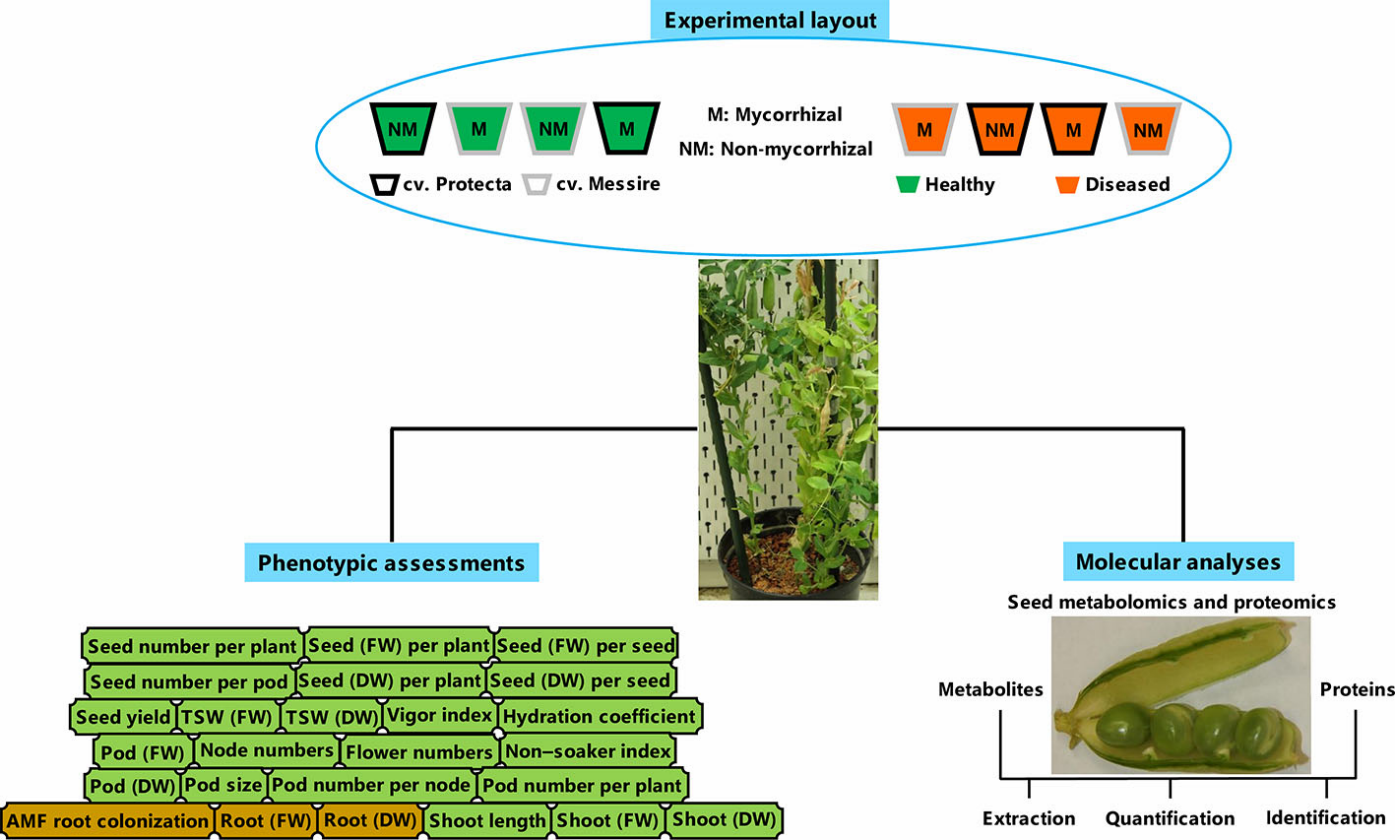
Schematic summary of experimental layout, molecular, and phenotyping analyses. FW, fresh weight; DW, dry weight.

In line with processes carried out before (Ranjbar Sistani et al., 2017) and according to Begum et al. (2001), the disinfected pea seeds (ethanol 95%) were washed by ultrapure water and then, were kept (20 min) in bleach 5% and rinsed by ultrapure water several times. Finally, the soaked seeds (4 h) were considered for planting (Begum et al., 2001). The pre-germinated pea seeds during 3 days on the sterile perlite-vermiculate substrate were planted in pots containing sterilized soil with the following chemical properties: K_2_PO 120 mg/L, N 7 mg/L, pH 5.6 and P 13 mg/L (Desalegn et al., 2016). The pots were kept under the following conditions: 600 µmol m^−2^ s^−1^ lighting (14-h day/10-h night), humidity 60%–70%, and 22°C day/16°C night (Ludidi et al., 2007; Larrainzar et al., 2014). Treatments consisting of tolerant [cultivar Protecta (cv. Pr)] and susceptible [cultivar Messire (cv. Me)] genotypes, symbiont (M = mycorrhizal, NM = non˗mycorrhizal) and pathogen (infected/diseased = I and uninfected/healthy = U) were arranged in pots. Four plants per biological replicate (three biol. replicates) and two technical replicates per each treatment were considered. For metabolomics, proteomics, and phenotypic evaluations of seed, a hundred seeds per each biological replicate were considered. A modified recipe of B&D (Broughton and Dilworth, 1970) nutrient solution (KNO_3_ 1011.03 ppm, MgSO_4_·7H_2_O 61.65 ppm, ZnSO_4_·7H_2_O 0.14 ppm, Fe-citrate 2.63 ppm, MnSO_4_·H_2_O 0.17 ppm, CaCl_2_ 147 ppm, CoSO_4_·7H_2_O 0.028 ppm, K_2_SO_4_ 43.5 ppm, Na_2_MoO_4_·2H_2_O 0.024 ppm, CuSO_4_·5H_2_O 0.05 ppm and H_3_BO_3_ 0.12 ppm) was used for mycorrhizal treatments and B&D solution containing KH_2_PO_4_ (68 ppm) was applied for non-AMF treatments (Desalegn et al., 2016). An overview of the experimental setup can be found in **Figure 1**.

### AMF Inoculation

Commercial mycorrhizal inoculum SYMBIVIT^®^ (Symbiom Ltd., Czech Republic, www.symbiom.cz) containing five naturally occurring Glomerales species: *Claroideoglomus etunicatum, C. claroideum, Rhizophagus irregularis, Funneliformis geosporus*, and *F. mosseae*, was used. For mycorrhizal symbiosis establishment, 5 g of mycorrhizal inoculant (Horii and Ishii, 2014) according to company recipe and a modified method of Al-Karaki and Clark (1999) was added into each planting hole. Seeds were placed on the top of the inoculant (Al-Karaki and Clark, 1999).

### Pathogen Inoculation and Disease Severity Estimation

According to our previous study (Ranjbar Sistani et al., 2017) and in detail: the prepared pathogen (*D. pinodes*) by Rubiales Lab in Spain was multiplied on Potato Dextrose Agar (PDA) and incubated upon 12-h photoperiod and 22°C (Davidson et al., 2012). Incubated colonies of pathogen after a week was used to make fungal suspension by adding Milli-Q (ultrapure) water and scratching of colonies upon sterile conditions (Zimmer and Sabourin, 1986; Carrillo et al., 2013). The autoclaved cheesecloth was applied to filter the prepared suspension and the filtered pathogen suspension with concentration 3 × 10^5^ spores/ml was selected for inoculation (Zimmer and Sabourin, 1986; Carrillo et al., 2013). The fungal suspension plus TWEEN 20 (120 μl/100 ml) was handled to inoculate the pea seedlings after 20 days of planting separately from healthy (uninfected) treatments that were sprayed by a mixture of water and TWEEN 20 (Carrillo et al., 2013). Then, inoculated seedlings were kept under transparent covers during 48 h (Carrillo et al., 2013; Okorska et al., 2014) and after this period, infected seedlings were set apart for one week (12-h photoperiod, 21 ± 2°C) (Garry et al., 1998). Afterward, all seedlings were arranged as described in *Performing Experiments and Planting*, the aerial parts of infected pea plants by *D. pinodes* were regularly and periodic investigated by using a USB digital microscope (25X-400X, BMSCI, Japan) to image and computing the lesions (Ranjbar Sistani et al., 2017). The captured dimensions of lesions were applied to calculate the area of lesions and finally disease severity assay (Hwang et al., 2006; Carrillo et al., 2013). To complete the disease severity assessments, the infection rate of seeds in diseased pea plants was specified through the paper towel and PDA methods (Xue et al., 1996; Gaurilˇcikien˙e et al., 2012; Mahmoud et al., 2013) besides the area of lesions.

### Root Colonization Assay

#### Sample Preparation

The pea plants were harvested when pods and seeds were ripe (BBCH 81-88). The root samples were prepared for investigation of mycorrhizal root colonization when the seeds were matured (Al-Karaki and Clark, 1999; Horii and Ishii, 2014). According to the modified method of Jin et al., 2013, soil particles were removed from the root samples using tap water, washed again with Milli-Q (ultrapure) water, and then dried with a paper towel. For each pot, subsamples of about 0.1–0.2 g were separated from the root system, freshly weighted (FW1) and immediately the fresh weight of remaining root was recorded (FW2) besides determining of the dry weight of that larger sample (DW2) [(Giovannetti and Mosse, 1980; Plant & Mycorrhiza Root Lengths ^2^)]. The small subsamples of pea roots were boiled (10% KOH, 15 min) and the boiled root samples were washed by using water (Vierheilig et al., 1998). For staining, the cleaned root samples were put in boiling ink-acetic acid (5%, 3 min), and then, the stained root samples were washed (20 min) with acidified water by acetic acid (Vierheilig et al., 1998).

#### Mycorrhizal Colonization Assay

The mycorrhizal colonization of *P. sativum* roots was studied based on the gridline intersect method (Giovannetti and Mosse, 1980). On average, 20 stained root samples per pot were investigated. The count of intersections between lines, NM roots (R1), and M roots (R2) were recorded (INVAM^2^). The percentage of root colonization by AMF was calculated per each root sample by the following formula (Giovannetti and Mosse, 1980; Jin et al., 2013; INVAM^2^):

$$\text{AMF root colonization}=\frac{\text{ Total mycorrhizal intersections}}{\text{Total root intersections}}\times 100$$

Moreover, the dry weight of the small subsample (DW1) was calculated by the following equation: DW1 = FW1 × DW2/FW2 (INVAM^2^). For the larger subsample that was not stained, the mycorrhizal root length (R3) was estimated [R3 = R2 × DW2 / DW1] (INVAM^2^). Total mycorrhizal root length (TMRL) and total plant root length (TPRL) were determined by the following formulas: TMRL = R2 + R3; TPRL = R1 + R3 (INVAM^2^).

### Investigation of Physical Properties and Yield of Pea Seeds

In compliance with prior work (Ranjbar Sistani et al., 2017), the total weight of pea seeds per each pot was recorded and the seed yield (kg/ha) formula that was mentioned in Sajid et al. (2012) work was adapted to determine the seed yield per treatment (kg pot^−1^). The physical quality of pea seeds was evaluated through quantification of absorption and hardness rates of pea seeds per treatment that were kept in water (16 h) and then were assessed by hydration coefficient (%) and non-soakers (%) formulas (Abdelgani et al., 1999). The vigor index of pea seeds was quantified based on seed germination rate and seedling length measurements by using rolled paper towels (Abdul Baki and Anderson, 1973; Farrag and Moharam, 2012).

### Metabolomics and Proteomics Studies of *P. sativum* Seeds

#### Sample Preparation

Mature seeds of *P. sativum* were freeze-dried by liquid nitrogen and then were coldly milled by Retsch mixer mill MM 400 (Retsch, Germany) (Ranjbar Sistani et al., 2017).

#### Extraction, Digestion, Desalting, and Mass Spectrometry of Seed Proteins

The seed proteins were extracted according to an adapted protocol of Wienkoop et al. (2008): 1.5 ml of extraction buffer [PMSF (1 mM), sucrose (0.7 M), EDTA (5 mM), Milli-Q water, PVPP (1% w/v), DTT (5 mM), and 50 mM Tris-HCl (pH 7.5)] was added to 50 mg of resulting powder from seed milling and then homogenization was completed by adding 1.5 ml Roti^®^-Phenol (Carl Roth GmbH, Karlsruhe, Germany). After that, the shaken mixtures (30 min, 4°C) were centrifuged (4°C, 4,000 × g, 30 min) and the resulting supernatants were precipitated with acetone at −20°C overnight. Then, the supernatant was centrifuged (4°C, 4,000 × g, 15 min), and then air-dried resulting pellet was dissolved with urea buffer [urea (8 M), HEPES (50 mM, pH 7.8)]. After protein concentration determination (Bradford, 1976), the protein pre-digestion (150 μg), was done by adding endoproteinase Lys-C sequencing grade (30°C, 5 h; Roche, Germany) followed by trypsin digestion, adding Poroszyme immobilized trypsin beads (5 μl; Applied Biosystems, Germany) and overnighting incubation in a hybridization oven (37°C). Afterward, digested proteins were desalted by using C18-SPEC 96-well plates (Agilent technologies) followed by an arrangement of desalting output in a vacuum centrifuge concentrator and eventually, storage at −80°C. For mass spectrometry of *P. sativum* seed, dried samples (1 μg per sample) were dissolved in a mixture containing formic acid (0.1%) and acetonitrile (2%) and were analyzed by Orbitrap Elite Hybrid Ion Trap-Orbitrap Mass Spectrometer (Thermo Fisher Scientific, Bremen, Germany) that was connected to a 1D nano LC (UltiMate 3000, Thermo Fisher Scientific) through loading into a column (EASY-Spray, PepMap C18, >2 µm particles, 100 Å pore size, 15 cm × 50 µm ID) (PepMap RSLC, Thermo scientific). MS settings were: top 20 MS2 scans (CID activation), charge state screening enabled with the rejection of unassigned and +1 charge states, scan range (350–1,800 m/z), repeat (count and duration: 1 and 30 s), required signal threshold (Min. 1,000) and exclusion (list size and duration: 500 and 60 s).

#### Quantification and Identification of Pea Seed Proteins

The MaxQuant (version 1.6.0.16, Cox and Mann, 2008) was used with the following parameters: label-free quantification (MS/MS, large and min ratio: 2), missed cleavages (max: 2), min peptide length (unspecific search): 6, main and first search peptide tolerances: 4.5, 20 ppm respectively, FDR and PSM: 0.01, match tolerances (centroid: 7.5 and isotope: 2 ppm), variable modifications (max: 5) and activated decoy mode revert. For a sequence database in the Andromeda search engine (Cox et al., 2011), a constructed FASTA protein database (Desalegn et al., 2016; Turetschek et al., 2016) was used. Fasta, mercator, and MS data files with the identifier PXD006617 were stored in the PRIDE database of ProteomeXchange Consortium (Vizcaíno et al., 2016).

#### Extraction of Seed Secondary Metabolites and Seed Metabolomics by NanoESI LC-MS/MS

A modified protocol of De Vos et al. (2007) was applied for seed metabolomics as described before (Ranjbar Sistani et al., 2017): the mixed ground pea seed (100 mg) with 1 ml methanol (80%) were arranged in a filled ultrasonic bath with ice and water (10 min) followed by centrifugation (10 min, 21,000 × g). The resulting supernatant was dried in a vacuum centrifuge concentrator and then each dried sample was mixed with 50 μl [methanol (50%) + FA (0.1%)] and was centrifuged. After that, the reserpine (3μl, Sigma-Aldrich) was added to each sample which was diluted (1:10) by methanol (5%) in FA (0.1%) and were centrifuged again. For mass spectrometry, 20 μl of each supernatant was used. The NanoESI LC-MS/MS was carried out by using LTQ-Orbitrap XL Hybrid Ion Trap-Orbitrap Mass Spectrometer (Thermo Fisher Scientific, Germany) with HPLC column and same parameters setting to proteomics analysis except for required signal (min 50,000) and scan range (130–1,800 m/z).

#### Profiling, Quantification, and Identification of Seed Metabolites

Annotation, quantification, and identification of the extracted metabolites were carried out as previously described (Ranjbar Sistani et al., 2017), with some changes: for identification and quantification of metabolites, Xcalibur (version 2.3.26, Thermo Fisher Scientific Inc.) was used for conversion of mass spectrometry output (RAW files) to CDF files. The resulting CDF files were used as input of MET-COFEA (Zhang et al., 2014). Then, the output files of MET-COFEA were loaded into the MET-XAlign (Zhang et al., 2015) tool. In parallel, the created mzXML files from the conversion of RAW files by MassMatrix (version 3.9, Case Western Reserve University^3^) were processed by using ProtMAX 2012_rev.2.14 (Egelhofer et al., 2013) as well as a spectral survey (Wang et al., 2016) and peak integration (Xcalibur 2.2, Thermo Scientific). The resulting data from MET-XAlign and ProtMAX outputs were used to complete the metabolite identification by using HMDB^4^ (Wishart et al., 2018), METLIN^5^ (Smith et al., 2005; Guijas et al., 2018), KNApSAcK family^6^ (Afendi et al., 2012). The metabolite candidates were finalized through literature reviews.

### Statistical Data Analysis

The normality of data was checked by using the Shapiro-Wilk test and to find the significant differences in treatments, ANOVA with Tukey HSD (P-value < 0.05) as a *post hoc* test, and the Kruskal-Wallis test were applied by using STATGRAPHICS Centurion 18^7^. To study the major impacts of factors (microsymbiont, cultivar, and pathogen) and their interactions for phenotypic data, multifactor (three-way) ANOVA plus Tukey HSD procedure (P-value < 0.05) was performed in STATGRAPHICS Centurion 18^7^. To analyze the correlations among all phenotypic variables, Pearson correlation coefficients through STATGRAPHICS Centurion 18^7^ were calculated. Only proteins and metabolites that were reproducibly detected in all three biol. replicates (averages of two technical replicates per biological replicate) of at last one treatment were considered for quantification (Ranjbar Sistani et al., 2017). Filling of missed values base on a prior distribution followed by z-transformation was performed for independent component analysis (ICA) and cluster analysis by using COVAIN (Sun and Weckwerth, 2012) (Updated 2019; MATLAB R2017a, The MathWorks, Inc., Natick, Massachusetts, United States.). The q-values were calculated using an online Shiny application^8^ of q-value R package^9^ (Storey, 2002; Storey, 2003; Storey and Tibshirani, 2003; Storey et al., 2004; R Core Team, 2013).

## Results

### Mycorrhizal Colonization of *P. sativum* Roots

The mean mycorrhizal colonization in roots of inoculated pea plants was ranged between 64.78% and 80.13% (**Table S1**). Although the mean AMF colonization between two cultivars was not significantly different, cultivar Protecta (cv. Pr) indicated higher colonization (11%) compared to cultivar Messire (cv. Me) (**Table S1**). Similarly, mycorrhizal colonization was not significantly affected by pathogen infection (**Table S1**), however, uninfected pea plants were more colonized (12%) by AMF than infected plants (**Table S1**). Although, TMRL and TPRL were not significantly different between the two cultivars, cv. Pr had higher root lengths (mycorrhizal-colonized and not) in comparison with cv. Me (**Table S1**). Root (FW and DW) were strongly and positively correlated with TMRL and TPRL (**Table S2**). Also the symbiont factor showed a significant impact on root fresh and dry weights (**Table 1**).

**Table 1:** Three-way ANOVA of main effects of AMF, pathogen, cultivar type, and their interactions impact on above–belowground yield and growth components of *P. sativum*.

SOV	Effects						Interactions							
	S		C		P		S × C		S × P		C × P		S × C × P	
	Symbiont (AMF)		Cultivar		Pathogen									
	MS	F	MS	F	MS	F	MS	F	MS	F	MS	F	MS	F
Number of flowers	337.50	3.85	2440.17	27.83***	640.67	7.31*	294.00	3.35	1.50	0.02	181.50	2.07	96.00	1.10
Number of nodes	0.00	1.30	0.00	7.61*	0.01	11.94**	0.00	0.19	0.00	0.75	0.00	0.47	0.00	0.07
Pod number per plant	42.67	3.52	60.17	4.96*	486.00	40.08***	73.50	6.06*	0.67	0.05	28.17	2.32	28.17	2.32
Pod number per node	0.00	0.22	0.02	2.89	0.00	0.16	0.00	0.02	0.00	0.08	0.00	0.06	0.00	0.11
Pod size (cm)	0.91	11.14**	0.78	9.53**	0.28	3.41	0.00	0.01	0.00	0.02	0.00	0.03	0.00	0.03
Pod weight (g)	0.27	4.75*	0.26	4.63*	1.18	20.76***	0.03	0.49	0.08	1.42	0.03	0.46	0.07	1.30
Shoot–FW (g)	3991.42	11.64**	1930.27	5.63*	7629.53	22.25***	7.96	0.02	238.84	0.70	135.89	0.40	175.69	0.51
Shoot–DW (g)	0.01	3.94	0.01	5.98*	0.05	23.9***	0.00	0.42	0.00	1.19	0.00	0.28	0.00	0.86
Shoot length (cm)	0.00	1.33	0.13	87.94***	0.03	18.87***	0.00	0.02	0.00	0.22	0.00	0.07	0.00	0.93
Root–FW (g)	5.06	144.42***	1.35	38.62***	0.56	16.03**	0.01	0.37	0.00	0.03	0.23	6.5*	0.04	1.07
Root–DW (g)	2.68	142.23***	0.31	16.53***	0.11	6.06*	0.00	0.04	0.02	0.92	0.05	2.61	0.00	0.00
Seed number per plant	37.50	0.35	121.50	1.14	4428.17	41.42***	308.17	2.88	253.50	2.37	228.17	2.13	228.17	2.13
Seed number per pod	0.21	1.83	0.11	0.96	0.19	1.64	0.01	0.12	0.24	2.07	0.00	0.00	0.00	0.01
Seed (FW) per seed	0.03	45.99***	0.01	21.73***	0.00	0.70	0.00	2.86	0.00	1.79	0.00	3.10	0.00	0.00
Seed (DW) per seed	0.01	32.19***	0.00	0.33	0.00	0.53	0.00	4.16	0.00	4.72*	0.00	0.39	0.00	3.74
Seed (FW) per plant	147.78	6.9*	0.46	0.02	693.30	32.39***	17.04	0.80	109.69	5.12*	56.87	2.66	146.74	6.85*
Seed (DW) per plant	30.40	3.72	20.83	2.55	251.53	30.76***	5.56	0.68	47.73	5.84*	17.72	2.17	22.08	2.70
TSW–FW (g)	25711.50	45.99***	12149.40	21.73***	391.06	0.70	1598.91	2.86	1003.08	1.79	1732.61	3.10	0.53	0.00
TSW–DW (g)	6188.73	32.19***	62.84	0.33	102.84	0.53	799.28	4.16	906.83	4.72*	75.26	0.39	718.44	3.74
Seed yield (kg)	1.76	6.9*	0.01	0.02	8.25	32.39***	0.20	0.80	1.30	5.12*	0.68	2.66	1.75	6.85*
Non–soaker index	0.51	3.06	0.90	5.45*	0.12	0.72	0.09	0.56	0.04	0.25	0.04	0.22	0.02	0.14
Hydration coefficient	0.33	0.12	6.56	2.33	0.06	0.02	1.52	0.54	0.01	0.00	0.01	0.00	0.01	0.00
Vigor index	0.00	0.02	0.34	3.49	3.09	31.67***	0.13	1.32	0.00	0.00	0.13	1.34	0.12	1.18
Mycorrhizal root colonization			164.30	2.11	189.76	2.44					71.72	0.92		
TMRL (cm)			44662.40	2.26	101.46	0.01					45124.50	2.28		
TPRL (cm)			42285.10	2.16	0.38	0.00					47894.60	2.44		
Area of lesions	0.08	22.09**	0.00	0.44			0.00	0.02						
Seed infection level	0.20	27.42***	0.25	34.2***			0.01	0.78						

SOV, source of variation; MS, mean square; F, F-ratio; S, symbiont; C, cultivar; P, pathogen; TSW, thousand seed weight; DW, dry weight; FW, fresh weight; TMRL, total mycorrhizal root length; TPRL, total plant root length. Asterisk symbols show the significant [* (p < 0.05), ** (p < 0.01), and *** (p < 0.001)] impacts.

### AMF Impact on Yield Components, Growth Parameters, and Seed Physical Properties

The following results are according to multifactor ANOVA to determine the variability of phenotypic parameters under the influence of three factors (symbiont, cultivar, and pathogen) besides comparison of treatments (M vs. NM) per each phenotypical variable. Although symbiont (AMF) factor had a significant effect on pod size, pod weight, seed (FW and DW) per seed, seed (FW) per plant, thousand seed weight TSW (FW and DW), seed yield, root (FW and DW), and shoot (FW) (**Table 1**), the yield components showed no significant changes between M and NM treatments (**Tables 2** and **3**). Cultivar and pathogen factors separately indicated significant effects on pod number per plant, pod weight, shoot length, number of flowers, number of nodes, shoot (FW and DW), and root (FW and DW) (**Table 1**). Cultivar and symbiont factors individually showed a significant influence on pod size, seed (FW) per seed, and TSW (FW) (**Table 1**). Also, the pathogen factor had a significant impact on seed (FW) per plant, seed yield, seed (DW) per plant, and seed number per plant (**Table 1**). Among seed physical properties, cultivar and pathogen factors separately demonstrated a significant effect on non-soaker and vigor indexes respectively (**Table 1**). Among yield components, symbiont, cultivar, and pathogen factors had significant effects separately on pod weight while the triple interaction effect of these factors on seed (FW) per plant and seed yield were significant (**Table 1**). Additionally, a significant and positive correlation was observed between seed number per plant and mycorrhizal colonization percentage while the none-soaker index was significantly and negatively correlated with AMF colonization, TMRL, and TPRL (**Table S2**). Although the majority of yield components showed no significant changes between M and NM treatments (**Tables 2** and **3**) but the seed (FW and DW) per seed and TSW (FW and DW) in cv. Pr were significantly enhanced in M plants vs. NM under healthy and diseased conditions (**Table 2**). Furthermore, root (FW and DW) were significantly enhanced in mycorrhizal (M) vs. non˗mycorrhizal (NM) plants upon healthy and diseased across both cultivars (**Table 3**).

**Table 2:** The yield components and seed physical properties of pea plants upon different treatments and conditions.

Treatment	Seed yield (kg)	Seed (FW) per seed (g)	Seed (DW) per seed (g)	Seed (FW) per plant (g)	Seed (DW) per plant (g)	TSW–FW (g)	TSW–DW (g)	Seed number per plant	Seed number per pod	Hydration coefficient	Non–soaker index	Vigor index
**MeI**	2.33 ± 0.29 a	0.36 ± 0.01 a	0.24 ± 0.01 abc	21.33 ± 2.67 a	14.38 ± 1.65 ab	361.64 ± 13.65 a	243.78 ± 8 abc	60.00 ± 5.97 abc	2.69 ± 0.20 a	4.01 ± 0.97 a	2.16 ± 0.23 a	0.67 ± 0.18 a
**MeMI**	3.12 ± 0.29 ab	0.40 ± 0.01 ab	0.24 ± 0.01 abc	28.65 ± 2.67 ab	16.69 ± 1.65 abc	398.14 ± 13.65 ab	241.12 ± 8 abc	55.67 ± 5.97 ab	2.62 ± 0.20 a	4.22 ± 0.97 a	1.85 ± 0.23 a	0.97 ± 0.18 ab
**MeU**	3.24 ± 0.29 abc	0.36 ± 0.01 a	0.22 ± 0.01 ab	29.67 ± 2.67 abc	18.23 ± 1.65 abc	357.92 ± 13.65 a	219.95 ± 8 ab	81.33 ± 5.97 bcd	2.65 ± 0.20 a	3.90 ± 0.97 a	1.80 ± 0.23 a	1.38 ± 0.18 ab
**MeMU**	3.89 ± 0.29 bc	0.42 ± 0.01 abc	0.26 ± 0.01 c	35.65 ± 2.67 bc	22.35 ± 1.65 bc	419.69 ± 13.65 abc	263.76 ± 8 c	76.33 ± 5.97 bcd	3.00 ± 0.20 a	4.23 ± 0.97 a	1.78 ± 0.23 a	1.40 ± 0.18 ab
**PrI**	2.74 ± 0.29 ab	0.41 ± 0.01 ab	0.22 ± 0.01 ab	25.16 ± 2.67 ab	13.68 ± 1.65 a	407.60 ± 13.65 ab	221.60 ± 8 ab	45.00 ± 5.97 a	2.49 ± 0.20 a	5.57 ± 0.97 a	1.76 ± 0.23 a	1.04 ± 0.18 ab
**PrMI**	2.10 ± 0.29 a	0.48 ± 0.01 c	0.26 ± 0.01 c	19.22 ± 2.67 a	10.23 ± 1.65 a	476.16 ± 13.65 c	263.91 ± 8 c	67.33 ± 5.97 abcd	2.54 ± 0.20 a	4.85 ± 0.97 a	1.32 ± 0.23 a	0.78 ± 0.18 a
**PrU**	3.25 ± 0.29 abc	0.37 ± 0.01 a	0.21 ± 0.01 a	29.77 ± 2.67 abc	17.13 ± 1.65 abc	369.31 ± 13.65 a	212.57 ± 8 a	91.00 ± 5.97 d	2.49 ± 0.20 a	5.44 ± 0.97 a	1.68 ± 0.23 a	1.77 ± 0.18 b
**PrMU**	4.61 ± 0.29 c	0.46 ± 0.01 bc	0.26 ± 0.01 bc	42.26 ± 2.67 c	23.16 ± 1.65 c	464.32 ± 13.65 bc	257.58 ± 8 bc	88.00 ± 5.97 cd	2.91 ± 0.20 a	4.69 ± 0.97 a	1.28 ± 0.23 a	1.78 ± 0.18 b

Me, cv. Messire; Pr, cv. Protecta; M, mycorrhizal; NM, non-mycorrhizal; I, infected (diseased); U, uninfected (healthy). Values illustrate the means ± standard error from 12 pea plants per treatment. Different letters per column indicate significant (Tukey HSD test, p < 0.05) differences among treatments.

**Table 3:** The above–belowground growth parameters in different treatments of *P. sativum* under varied conditions.

Treatment	Number of flowers	Number of nodes	Pod number per node	Pod number per plant	Pod size (cm)	Pod weight (g)	Shoot–FW (g)	Shoot–DW (g)	Shoot length (cm)	Root–FW (g)	Root–DW (g)
**MeI**	38.00 ± 5.41 ab	0.39 ± 0.01 ab	0.27 ± 0.05 a	24.00 ± 2.01 abc	5.45 ± 0.17 ab	2.23 ± 0.14 a	37.67 ± 10.69 a	1.44 ± 0.03 a	1.74 ± 0.02 a	0.81 ± 0.11 a	0.75 ± 0.08 a
**MeMI**	34.00 ± 5.41 a	0.41 ± 0.01 ab	0.29 ± 0.05 a	21.33 ± 2.01 ab	5.79 ± 0.17 ab	2.38 ± 0.14 ab	63.21 ± 10.69 abc	1.47 ± 0.03 ab	1.75 ± 0.02 a	1.71 ± 0.11 b	1.46 ± 0.08 cd
**MeU**	38.33 ± 5.41 ab	0.42 ± 0.01 ab	0.25 ± 0.05 a	29.00 ± 2.01 abcd	5.65 ± 0.17 ab	2.61 ± 0.14 ab	69.47 ± 10.69 abc	1.53 ± 0.03 ab	1.8 ± 0.02 ab	1.24 ± 0.11 ab	1.03 ± 0.08 ab
**MeMU**	43.33 ± 5.41 ab	0.43 ± 0.01 b	0.27 ± 0.05 a	30.00 ± 2.01 bcd	6.06 ± 0.17 b	2.75 ± 0.14 ab	93.21 ± 10.69 bc	1.55 ± 0.03 ab	1.82 ± 0.02 abc	2.27 ± 0.11 c	1.64 ± 0.08 d
**PrI**	41.67 ± 5.41 ab	0.37 ± 0.01 a	0.20 ± 0.05 a	19.33 ± 2.01 a	5.08 ± 0.17 a	2.20 ± 0.14 a	44.29 ± 10.69 ab	1.45 ± 0.03 a	1.87 ± 0.02 bcd	1.44 ± 0.11 b	1.05 ± 0.08 ab
**PrMI**	59.67 ± 5.41 abc	0.38 ± 0.01 ab	0.23 ± 0.05 a	28.00 ± 2.01 abcd	5.48 ± 0.17 ab	2.70 ± 0.14 ab	82.95 ± 10.69 abc	1.54 ± 0.03 ab	1.91 ± 0.02 cd	2.41 ± 0.11 c	1.79 ± 0.08 d
**PrU**	61.00 ± 5.41 bc	0.41 ± 0.01 ab	0.21 ± 0.05 a	33.00 ± 2.01 cd	5.27 ± 0.17 ab	2.93 ± 0.14 b	96.43 ± 10.69 bc	1.59 ± 0.03 b	1.96 ± 0.02 d	1.65 ± 0.11 b	1.16 ± 0.08 bc
**PrMU**	72.00 ± 5.41 c	0.41 ± 0.01 ab	0.20 ± 0.05 a	36.67 ± 2.01 d	5.67 ± 0.17 ab	2.99 ± 0.14 b	111.65 ± 10.69 c	1.6 ± 0.03 b	1.96 ± 0.02 d	2.43 ± 0.11 c	1.78 ± 0.08 d

Me, cv. Messire; Pr, cv. Protecta; M, mycorrhizal; NM, non-mycorrhizal; I, infected (diseased); U, uninfected (healthy). Values illustrate the means ± standard error from 12 pea plants per treatment. Different letters per column indicate significant (Tukey HSD test, p < 0.05) differences among treatments.

### Mycorrhizal Colonization Effect on Disease Severity

The disease severity of *P. sativum* plants infected by *D. pinodes* was determined by the evaluation of the seed infection level and area of lesions. Although the mean area of lesions between the two cultivars was not significantly different, cv. Pr had lower lesions area (**Table 4**) and significantly lower levels of seed infection (Tukey HSD test, p < 0.05, nearly 2 folds) compared with cv. Me (**Table 4**). We found that AMF treatment of cv. Me resulted in a significantly lower level (Tukey HSD test, p < 0.05, nearly 2 folds) of seed infection (**Table 4**). Also, the inoculation of AMF significantly decreased the mean area of lesions in both infected cultivars (**Table 4**).

**Table 4:** Quantified analysis of disease severity caused by *D. pinodes* upon different treatments.

Treatments	Seed infection level	Area of lesions
MeI	0.70 ± 0.05 c	1.05 ± 0.03 c
MeMI	0.40 ± 0.05 b	0.88 ± 0.03 ab
PrI	0.37 ± 0.05 ab	1.02 ± 0.03 bc
PrMI	0.16 ± 0.05 a	0.86 ± 0.03 a

Me, cv. Messire; Pr, cv. Protecta; M, mycorrhizal; NM, non-mycorrhizal; I, infected (diseased); U, uninfected (healthy). Values represent the means ± standard error from monitored seeds and area of lesions (leaves and stems) of 12 plants per treatment. Different letters per column indicate significant (Tukey HSD test, p < 0.05) differences among treatments.

### The Effect of AMF on Pea Seed Metabolites Under Infection Caused by *D. pinodes*

In total, 47 metabolites from 12 chemical classes and families were identified. The identified metabolites mostly belonged to glycerophospholipids, flavonoids, isoflavonoids, prenol lipids, carboxylic acids and derivatives, and organooxygen compounds (**Table S3A**). Glycerophosphoinositol phosphates (PIPs) as well as 2',4',5-Trihydroxy-7,8-[2-(1-methylethenyl) furo] isoflavone and lupinisoflavone A could not be assigned separately (due to identical chemical formula, retention time and m/z) and were therefore assigned as compound groups of PIPs and putative metabolites (*2',4',5-Tri_Lup_Isoflavon), respectively (**Table S3A**). Compared to our previous study, we increased our metabolite identification due to the additional use of the KNApSAcK family (http://kanaya.naist.jp/KNApSAcK_Family/) database. Eighty-seven percent of identified metabolites were significantly changed (ANOVA, Kruskal-Wallis, Tukey HSD tests, P-value < 0.05, and ≥ two-fold change) among treatments (M vs. NM of diseased plants across both cultivars) (**Table S3B**). Most of the metabolites that significantly increased in M vs. NM treatments were induced upon pathogen infection and mainly in cv. Pr (**Table S3B**). For example, (Z)-3-Oxo-2-(2-pentenyl)-1-cyclopenteneacetic acid, cyanidin 3-sophoroside 5-glucoside, luteone 7-glucoside, and stachyose were significantly enhanced in the seed metabolome of M vs. NM of cv. Pr under pathogen infection (**Table S3B**).

In contrast, L-2-Amino-3-(oxalylamino) propanoic acid (or L-3-Amino-2-(oxalylamino) propanoic acid), N-(Carbethoxyacetyl)-4-chloro-L-tryptophan, and vignatic acid A were only significantly accumulated in M vs. NM treatments of cv. Me against pathogen infection (**Table S3B**).

Most of the increased seed metabolites of M plants upon infection were carboxylic acids and derivatives (**Tables S3A, B**). Furthermore, some metabolites including 2,3-Dihydroxy-2,4-cyclopentadien-1-one, L-DOPA 3'-glucoside, soyasapogenol C and syoyualdehyde were significantly enhanced in M vs. NM of diseased treatments (**Table S3B**).

Independent of M or NM treatments, pathogen-infected versus healthy plants (I vs. U) showed a significant increase of malvidin 3-rutinoside-5-glucoside, peonidin 3-rhamnoside 5-glucoside, PI (20:5(5Z,8Z,11Z,14Z,17Z)/0:0), wistin, *2',4',5-Tri_Lup_Isoflavon, sativan, 6''-O-Malonylwistin, abscisic acid and kudzusaponin SA1 (**Table S3B**). In contrast, soyasapogenol C, syoyualdehyde, (Z)-3-Oxo-2-(2-pentenyl)-1-cyclopenteneacetic acid, 8-Galactopyranosyl-6-glucopyranosyl-4',5-dihydroxy-7-methoxyflavone, luteone 7-glucoside, N-Jasmonoylisoleucine and stachyose levels were significantly reduced in I vs. U treatments (**Table S3B**).

Among metabolites that showed a significant difference between two cultivars, 8-Galactopyranosyl-6-glucopyranosyl-4',5-dihydroxy-7-methoxyflavone, delphinidin 3-lathyroside 5-glucoside, (Z)-3-Oxo-2-(2-pentenyl)-1-cyclopenteneacetic acid, 6-O-b-D-Fructofuranosyl-2-deoxy-D-glucose, indole-3-acetamide, were remarkably enhanced in cv. Pr (**Table S3B**).

### Influence of AMF-Root Colonization on Pea Seed Proteome

Only proteins without missing values (LFQ intensities) across replicates of the same treatment were analyzed statistically. The resulting 1,325 proteins were functionally categorized by applying the Mercator pipeline (Lohse et al., 2014) and MapMan tool (Thimm et al., 2004) (**Table S4**). **Table S4** illustrates the comprehensive seed proteomics data including categorized proteins, protein descriptions, mapped functional classes, statistical analyses, and fold change (FC) ratio for all treatments. Overall, remarkable changes (ANOVA, Kruskal-Wallis, Tukey HSD test, and P-value < 0.05 and ≥ two-fold change) were found in 25% of mapped proteins by comparing M vs. NM under diseased and healthy conditions in both cultivars (**Table S4**).

**Figure 2:**
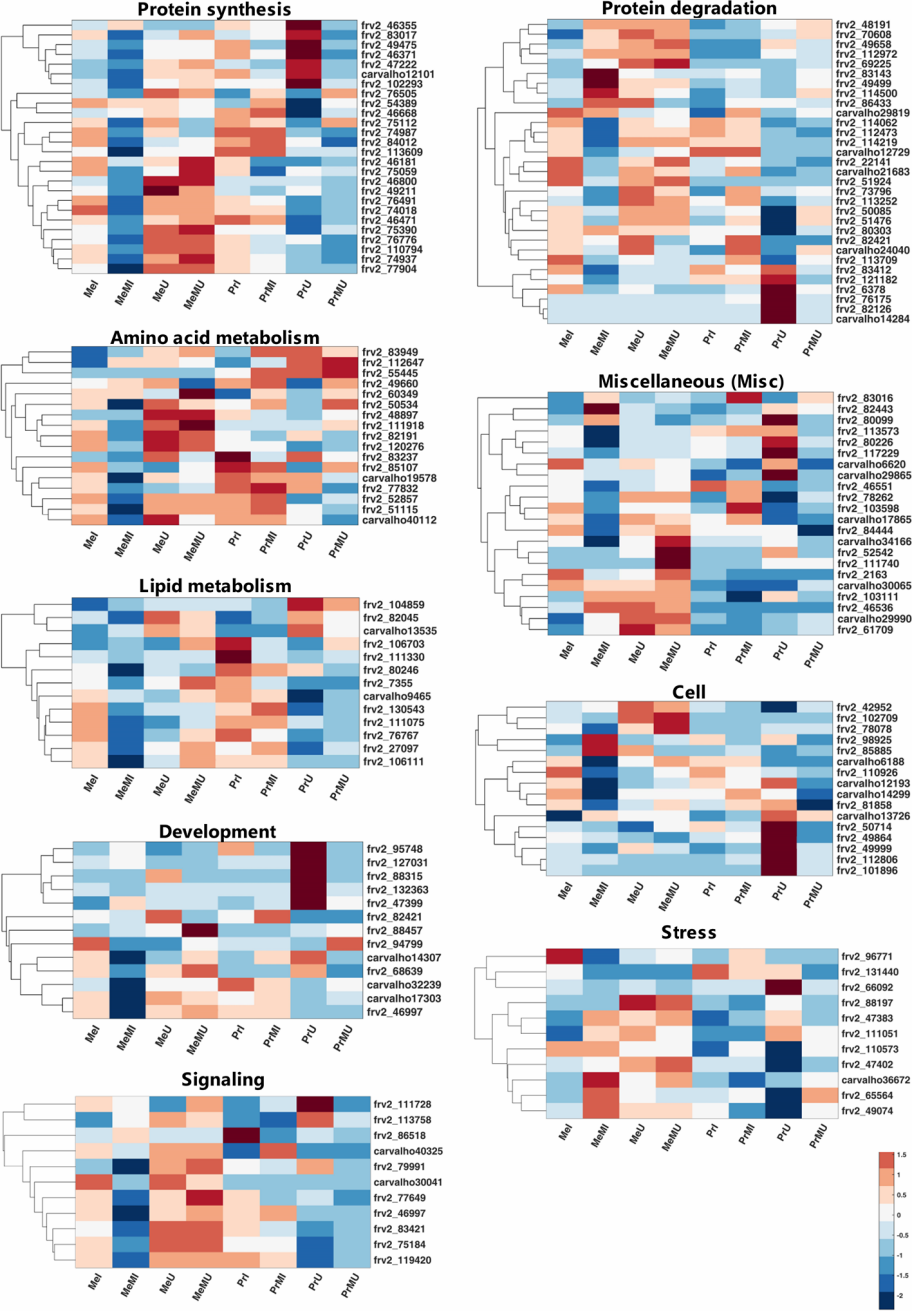
Clustered heatmaps of mapped functional categories which including ≥ 10 proteins of seed proteins significantly changed (Kruskal-Wallis; ANOVA, Tukey HSD test; p < 0.05 and ≥ two-fold change) in M compared with NM treatments upon I and U conditions across both cultivars. The heatmap cells are z-transformed of mean LFQ intensities per treatment. Me, cv. Messire; Pr, cv. Protecta; M, mycorrhizal; NM, non-mycorrhizal; I, infected (diseased); U, uninfected (healthy).

Among mapped functional categories that were significantly changed (P-value < 0.05 and ≥ two-fold change) in M vs. NM, the category of protein degradation had the highest number of proteins (**Table S4**). Most of the identified proteins were involved in protein degradation and thus several of those were found with significantly increased levels in M vs. NM treatments (**Table S4**). Among M vs. NM treatments under healthy conditions, protein degradation, protein synthesis, and stress categories in cv. Pr and plastid (PS) in cv. Me had the largest number of proteins changed upon M (**Table S4**). Protein functional categories such as amino acid metabolism (**Figure 2**; frv2_55445, frv2_85107, frv2_60349, frv2_112647, frv2_48897 and frv2_83949), miscellaneous (misc) (**Figure 2**; frv2_61709, frv2_111740, Q9FN08, frv2_46536, frv2_82443, P93479, frv2_103111, frv2_103598, frv2_78262, and frv2_83016) and redox (**Table S4**; frv2_125364, frv2_76239, frv2_47501, frv2_76239, frv2_77791, frv2_81620, and Q9FF55) showed a significant increase in M vs. NM upon disease and healthy conditions in both cultivars.

Under pathogen infection, several categories including DNA (**Table S4**; frv2_98925 and frv2_50256), mitochondrial electron transport (**Table S4**; frv2_77583, frv2_103383, frv2_81222, and frv2_53151), nucleotide metabolism (**Table S4**; frv2_87000, frv2_81447, frv2_112313, and frv2_48788), oxidative pentose phosphate (OPP) (**Table S4**; Q43848 and Q9SZE1), protein amino acid (aa) activation (**Table S4**; Q9ZPI1 and frv2_121810) and signaling (**Figure 2**; frv2_113758 and Q96453) were remarkably enhanced in M vs. NM of both cultivars. In contrast, the protein synthesis category (**Figure 2**; frv2_75059, frv2_46668, frv2_75112, frv2_54389, and frv2_76505) indicated a notable accumulation in M vs. NM exclusively under healthy conditions. In M vs. NM, the categories development (**Figure 2**; frv2_47399, frv2_88457, and frv2_94799) and PS (**Table S4**; Q43848, frv2_80442, frv2_77556, frv2_84713, and Q9SHE8) categories only in cv. Me besides C1-metabolism (**Table S4**; frv2_90362 and frv2_86521) and glycolysis (**Table S4**; frv2_114679, frv2_112199, frv2_47599, and Q88C93) uniquely in cv. Pr were significantly pronounced upon disease and health conditions.

Comparing of I vs. U upon M and NM treatments in both cultivars indicated that levels of several proteins engaged in amino acid metabolism, cell, DNA, lipid metabolism, metal handling, misc, mitochondrial electron transport, nucleotide metabolism, photosynthesis (PS), redox and signaling were significantly reduced while abundances of some proteins involved in proteins synthesis were remarkably enhanced (**Table S4**). Interestingly, some levels of proteins involved in protein degradation and stress categories were either notably accumulated or reduced in I vs. U (**Table S4**). Upon pathogen infection, the largest functional categories were protein synthesis (increased levels) and protein degradation (decreased levels) (**Table S4**). Some proteins assigned to fermentation, major CHO metabolism, and protein targeting were remarkably accumulated only in the seed proteome of NM treatments of both cultivars against pathogen infection. In contrast, some proteins of C1-metabolism, development, protein post-translational modification, TCA cycle, biodegradation of xenobiotics, and transport categories showed a significant reduction (**Table S4**).

Comparison of cultivars across all treatments including M and NM under disease and healthy conditions demonstrated that several proteins of N-metabolism, secondary metabolism, and signaling in cv. Me besides some proteins of amino acid metabolism and misc in cv. Pr were significantly (p < 0.05) and distinctively enhanced (**≥**2-fold) (**Table S4**). Moreover, several levels of proteins involved in cofactor and vitamin metabolism were significantly intensified in both cultivars of NM treatments against pathogen infection (**Table S4**). Numbers of identified proteins from OPP and protein post-translational modification classes in cv. Me in addition to some TCA cycle-related proteins in cv. Pr showed a significant enhancement in NM treatments upon pathogen infection (**Table S4**).

The proteins from main functional classes (including >2 proteins) with significantly increased fold change ratio (p < 0.05, FC **≥** 2) in M vs. NM under healthy and disease conditions of both cultivars are presented in **Figure S1**. Amino acid metabolism, misc and redox categories were only the main protein functional groups with enhanced FC significantly upon all paired comparisons of M vs. NM (**Figure S1A**). The most increased FC in M vs. NM belonged to protein synthesis in cv. Pr under healthy conditions (**Figure S1A**). Hormone metabolism and OPP had the highest increased FC in M treatments of cv. Me and cv. Pr respectively against pathogen infection (**Figure S1A**). Under pathogen infection, proteins associated with mitochondrial electron transport, nucleotide metabolism, OPP, protein amino acid (aa) activation and DNA were exclusively and largely accumulated in M vs. NM of both cultivars while, protein synthesis was uniquely promoted in M treatments of cultivars under healthy conditions (**Figure S1A**). When comparing M vs. NM, a significant increase of C1-metabolism, glycolysis, major CHO metabolism, and minor CHO metabolism were observed only in cv. Pr besides the notable accumulation of proteins related to development, PS, and TCA cycle individually in cv. Me were observed (**Figure S1A**). Protein degradation, protein synthesis, and stress functional classes were strongly increased in seed proteome of all I vs. U treatments in both cultivars, and on average among these categories, protein synthesis had the highest summed FC ratio (**Figure S1B**). Under disease conditions, engaged proteins in cell and biodegradation of xenobiotics illustrated noteworthy enhancement exclusively in cv. Pr while development, protein post-translational modification, and redox were highly intensified only in cv. Me (**Figure S1B**). The proteins included in signaling, stress, and TCA cycle were significantly promoted in cv. Pr compared with cv. Me upon M and NM treatments of both healthy and diseased (**Figure S1C**). Among these protein functional categories, stress-related proteins were highly increased in Pr vs. Me than proteins mapped in signaling and TCA cycle (**Figure S1C**).

### Integrated Analysis of Seed Metabolomics-Proteomics Data

**Figure 3:**
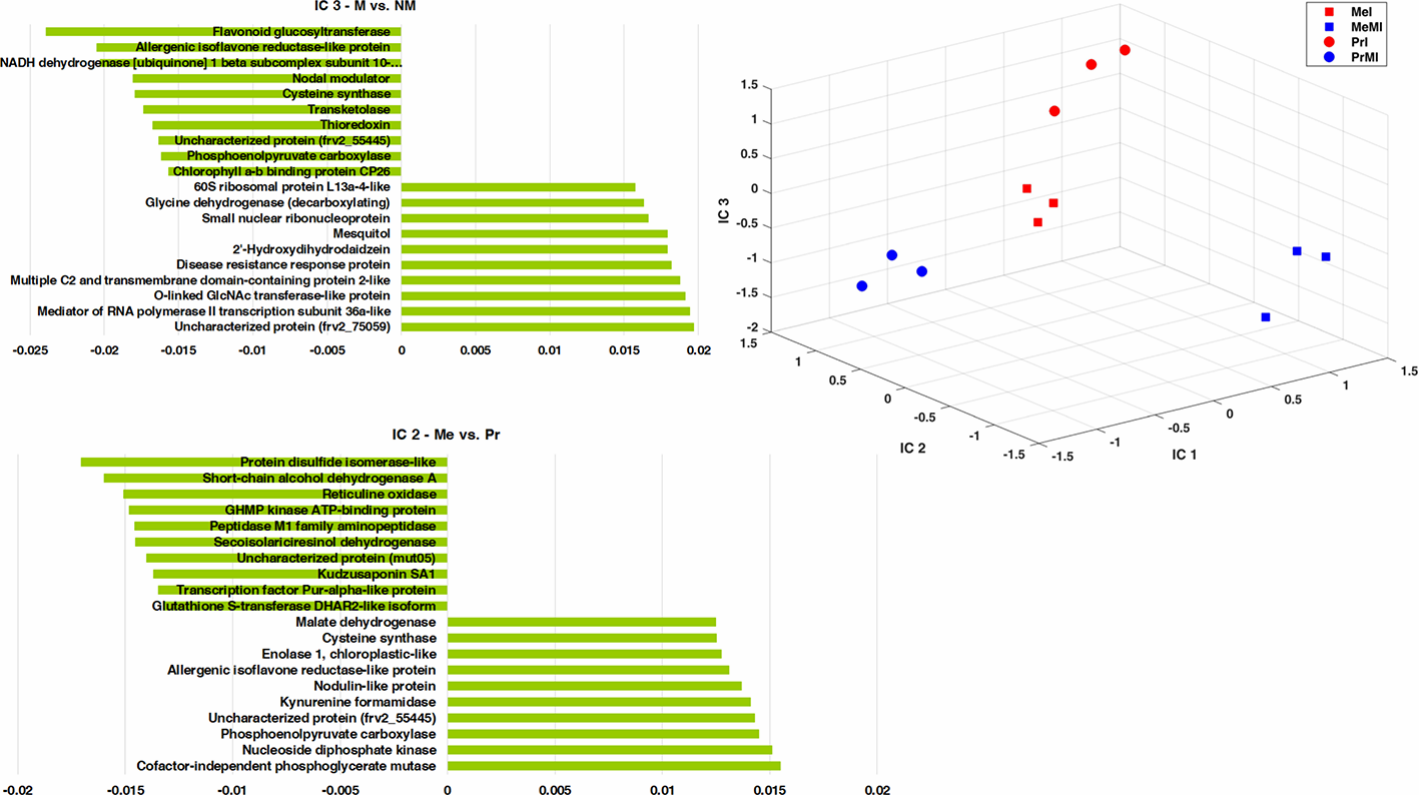
ICA of integrated seed metabolomics-proteomics data including remarkably changed (Kruskal-Wallis; ANOVA, Tukey HSD test; p < 0.05 and ≥ two-fold change) seed metabolites and proteins in M vs. NM upon diseased conditions of both cultivars. Numeric data were z-transformed. The loadings graphs illustrate the components with the top ten of the highest loadings (> 0.01 and < −0.01) on IC2 and IC3. Me, cv. Messire; Pr, cv. Protecta; M, mycorrhizal; I, infected (diseased).

To determine the metabolites and proteins with the highest impact on discrimination of treatments under stress conditions caused by pathogen, an ICA was performed (**Figure 3**). Only intensities of identified seed metabolites and proteins with significant alteration (ANOVA, Kruskal-Wallis, Tukey HSD tests, P-value < 0.05, and ≥ two-fold change) in M vs. NM upon diseased conditions of both cultivars were analyzed. The integrated seed metabolomics and proteomics data displayed distinctive separations of cultivars (Me vs. Pr) on IC2 and mycorrhizal symbiont (M vs. NM) on IC3 (**Figure 3**). **Figure 3** shows the ten highest positive and negative loadings responsible for these discriminations are depicted in separate bar plots.

Kudzusaponin SA1 and a Nodulin-like protein (frv2_125506) had considerable effects on the separation of Me vs. Pr in disease treatments (**Figure 3**). Some proteins like phosphoenolpyruvate carboxylase, allergenic isoflavone reductase-like protein, and cysteine synthase were highly involved in discrimination of M vs. NM besides Me vs. Pr upon diseased treatments (**Figure 3** and **Table S5**). An uncharacterized protein (frv2_55445) belonged to amino acid metabolism (degradation, arginine) had a high impact on the separation between both cultivars (Me vs. Pr) and M vs. NM in diseased treatments (**Figure 3** and **Table S5**). Two proteins (small nuclear ribonucleoprotein and mediator of RNA polymerase II transcription subunit 36a-like) mapped in RNA category were recognized with high influences on the separation of M vs. NM among diseased treatments (**Figure 3** and **Table S5**).

Cofactor-independent phosphoglycerate mutase (glycolysis) and protein disulfide isomerase-like (redox) had the highest impact on the separation of Me vs. Pr under diseased conditions (**Figure 3** and **Table S5**). Furthermore, an uncharacterized protein (frv2_75059) from protein synthesis class as well as flavonoid glucosyltransferase (secondary metabolism) indicated a strong effect on discrimination of M vs. NM upon diseased treatments (**Figure 3** and **Table S5**). Among disease treatments, two metabolites including 2'-hydroxydihydrodaidzein (isoflavonoid) and mesquitol (flavonoid) showed a remarkable impact on separating M vs. NM (**Figure 3** and **Table S5**).

**Figure 4:**
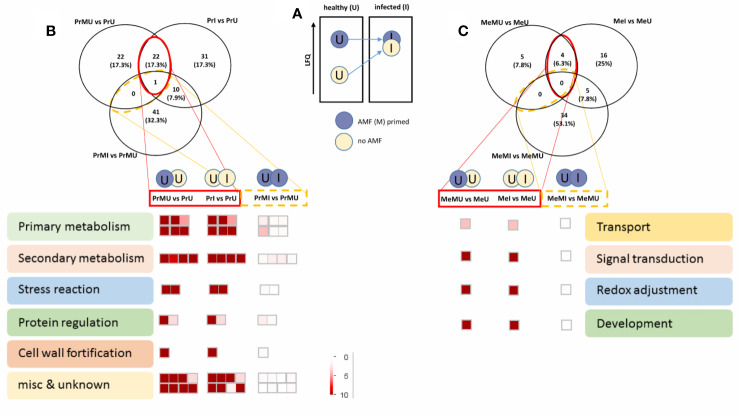
Overview of protein regulated pathways, involved in AMF priming. **(A)** Schematic concept of the AMF induced priming effect. Venn diagram and Mapman overview of selected overlapping proteins, significantly induced upon AMF treatment of healthy and infected non-symbiotic plants of cv. Protecta **(B)** and cv. Messire **(C)** (see also **Supplemental Table S6**). LFQ, Label-Free Quantification.

The data were then further reduced to focus on the most relevant protein groups potentially involved in Induced Systemic Resistance (ISR). For this, proteins that showed increased levels in M vs. NM of healthy plants overlapping with significantly increased levels upon pathogen infection of NM plants were selected for MapMan visualization (**Figure 4**). Names of the protein groups which were visualized, can be drawn from supplemental **Table S6** as they were in the same order. **Figure 4A** schematically shows the priming effect where protein levels of AMF symbiotic and healthy plants were induced compared to NM plants. During pathogen attack these protein levels also were increased in NM plants while they were not changed in infected M plants as their levels were already induced (primed) (**Figure 4**). Altogether, cv. Protecta shows a strong overlap of induced proteins (**Figure 4B** and **Table S6**) between healthy M treated and stressed non-symbiotic (PrI) plants while only one protein was also slightly further increased upon infection in M plants (MI). In contrast, only a few proteins (four proteins) were induced in healthy M plants of cv. Messire and overlapping with a stress response of non-symbiotic plants (**Figure 4C** and **Table S6**). The strongest responses of the seed metabolism of cv. Protecta (in terms of numbers) were proteins related to primary metabolism such as gluconeogenesis and amino acid synthesis and secondary metabolism-related proteins, including lipid and hormone (jasmonate) regulating proteins. In terms of fold changes, two proteins of yet unknown function revealed the highest induction (>100 fold) upon AMF symbiosis of healthy and after infection of NM Protecta plants (**Table S6**). The four proteins possibly involved in a priming effect in cv. Messire were a major intrinsic protein, a Polyvinylalcohol dehydrogenase-like protein (signaling), a Cytochrome b5-like protein, and a storage protein (legumin) (**Table S6**).

## Discussion

Thus far, major studies of mycorrhizal impacts on plants have been focused on some phenotypical components and/or improvement of nutritional properties in plants under optimal environmental growth conditions (Bethlenfalvay et al., 1994; Abdel-Fattah, 1997; Bethlenfalvay et al., 1997; Al-Karaki and Clark, 1999; Jin et al., 2013; Horii and Ishii, 2014; Kavitha and Nelson, 2014; Young et al., 2015; Abdel-Fattah et al., 2016). Even though there are increasing numbers of studies using omics technologies to investigate mycorrhizal symbiosis (Fontana et al., 2009; Pedone-Bonfim et al., 2013; Rebollo Couto et al., 2013; Laparre et al., 2014; Rivero et al., 2015; Saia et al., 2015; Bona et al., 2016; Larsen et al., 2016; Adolfsson et al., 2017; Li et al., 2017; Hill et al., 2018), the integration of several omics levels including metabolomics and proteomics with phenomics analyses is still a research gap. Also, most of this type of studies have concentrated on vegetative parts including shoot (Scheublin and van der Heijden, 2006), root (Schliemann et al., 2008; Song et al., 2015), leaf (Pedone-Bonfim et al., 2013; Schweiger et al., 2014; Desalegn et al., 2016; Adolfsson et al., 2017; Turetschek et al., 2017), and tuber (Lu et al., 2015). The current study investigates the mycorrhizal (M) impacts on above- and belowground yield and growth parameters integrating metabolomics (secondary metabolites) and proteomics analyses of seeds in two *P. sativum* cultivars in response to pathogen stress.

### AMF Efficiently Colonize *P. sativum* Roots and Influence Root Biomass

The successful colonization of plants by AMF is influenced by temperature, nutritional properties of soil, type of mycorrhizal symbiont, plant species, and light conditions (Smith and Smith, 1996; Johnson et al., 1997, as cited in Jansa et al., 2008). In this study, the high average AMF colonization (70%) of pea roots indicated a stable symbiosis between AMF species and the root system of *P. sativum* cultivars similar to our previous observations (Desalegn et al., 2016). Moreover, the considerable enhancement of root biomass in mycorrhiza symbiotic (M) plants compared with non-mycorrhizal (NM) plants in both cultivars and under disease stress prove the high impact of AMF on the root system as a plant growth-promoting symbiont in line with reported observations (Kavitha and Nelson, 2014; Oruru et al., 2017; Wang et al., 2018). Here, the remarkable increase of root biomass in M vs. NM plants besides significant positive correlation among root biomass (FW and DW) as well as between total mycorrhizal and plant root length (TMRL and TPRL) confirm the mutual connection between mycorrhizal root colonization and architecture of root system that was found before by Sinclair et al. (2014) in colonized roots of strawberry. However, to reach optimal AMF promoted crop productivity, the choice of AMF species that match with the various crops is determinative (Van Geel et al., 2016). Chen et al. (2017) have concluded that the application of AMF in a combined form of several mycorrhizal species is more effective compared with single inoculation on cucumber. This is important, as we can say that the AMF mixture, we used in this study, is effective even if we cannot distinguish between the putative differences in efficiency of the different species. Hence, although our approach is more close to nature, a differential analysis of the various AMF species remains a future task. Nevertheless, the mutual impact of AMF on root growth and vice versa, as indicated previously (Atkinson et al., 1994, as cited in Sinclair et al., 2014), corroborates our results for both pea cultivars. Further studies on AMF species and inoculum composition may further increase growth-promoting efficiency. Interestingly, the lower TMRL and TPRL in cv. Messire than cv. Protecta related not only to different root structures but also indicate the dissimilar response of the two cultivars to mycorrhizal root colonization by the same AMF species. Similarly, we previously observed an individual cultivar root-microsymbiont colonization pattern between cv. Messire and Protecta when inoculated with *Rhizobium* (Turetschek et al., 2017). Different AMF root colonization and root architecture were also observed in olive cultivars (Chatzistathis et al., 2013). Oruru et al., 2017 not only found the dissimilarities in AMF root colonization between modern and wild cultivars of cowpea, they also reviewed the differences among cultivars of tomato (Steinkellner et al., 2012), wheat (Tarawaya, 2003), maize (Njeru et al., 2013), and barley (Zhu et al., 2003). However, not much is known about the reason for and consequences of these root architectural differences in relation to symbiotic efficiency and hence, further root phenotyping (incl. molecular) studies will be needed. Finally, it is noteworthy that no significant negative effect of the pathogen on AMF colonization compared to healthy plants could be found regardless of cultivars. In contrast, our prior study (Ranjbar Sistani et al., 2017) showed significant reduction of *Rhizobium* density isolated from nodulated roots caused by *D. pinodes*. These results indicate that AMF root colonization is more stable under pathogen attack compared to *Rhizobium* symbiosis.

### Positive Effects of AMF Symbiosis on Growth, Yield, and Seed Physical Properties

In general, our results show that AMF as belowground symbionts also promote the growth of the above-ground parts of pea plants. Like in our previous study (Ranjbar Sistani et al., 2017), which focused on effects of bacterial (*Rhizobium*) symbiosis on pea seeds, here, we found that AMF had a significant impact on seed yield, seed (FW and DW) per seed, TSW (FW and DW), seed (FW) per plant, pod weight, and pod size. In earlier studies, the notable influence of AMF root colonization on seed yield enhancement (Cely et al., 2016; Adeyemi et al., 2017), seed weight per plant (Abdel-Fattah et al., 2016), pod weight (Abdel-Fattah et al., 2016; Adeyemi et al., 2017), and hundred-seeds weight (Kavitha and Nelson, 2014; Abdel-Fattah et al., 2016) has been observed. Specific proteins such as glutamine synthetase (N-metabolism), enolase (glycolysis), the protein disulfide isomerase (redox), malate dehydrogenase (TCA cycle), peptidyl-prolyl cis-trans isomerase (cell) were remarkably enhanced in seed proteome of M vs. NM pea plants. These proteins have been recognized previously as essential proteins during *Brassica campestris* seed development (Li et al., 2012). The data indicate that AMF influence seed growth and development of *P. sativum* cultivars.

Interestingly, Indole-3-acetamide (IAM), crucial for the synthesis of the auxin indole-3-acetic acid (IAA) (Mano et al., 2010), was reduced in our M treated plants. IAA is included in AMF symbiosis relation (Ludwig-Müller and Güther, 2007) and plant growth-regulation (Kögl and Kostermans, 1934; Went and Thimann, 1937). The role of AMF in promoting the synthesis of auxin and consequently enhancement of root-hair growth in *Poncirus trifoliate* was reported (Liu et al., 2018). The depletion of IAM upon AMF treatment might indicate that it was required for the formation of IAA, not detected in our study. Nevertheless, enhanced IAA production of seeds is not only of advantage for seed germination and development but also for microsymbiont attraction and interaction (Fu et al., 2015).

Strong genotype related impacts of AMF on pod weight in okra (Nwangburuka et al., 2012) have been reported earlier. Proteome analysis also revealed that secondary metabolism, general stress, and redox response and also proteins of the primary metabolism of amino acid synthesis and glycolysis are more enhanced in cv. Protecta compared to cv. Messire. Interestingly, most of the AMF responsive proteins of those metabolic pathways are also involved in pathogen defense.

### Belowground Mycorrhizal Symbiont Controls Seed Infection of the Aboveground Pathogen

The majority of studies associated with biocontrol and impact of mycorrhiza against plant pathogens are restricted to soil-borne and root fungal phytopathogens (Vierheilig et al., 2008). Here, the pathogen factor had significantly negative effects on aboveground parameters. The intensity of several proteins mapped for many functional classes including C1-metabolism, cell, development, DNA, metal handling, nucleotide metabolism, protein post-translational modification, redox, biodegradation of xenobiotics, signaling, TCA cycle, and transport were considerably reduced under stress caused by the pathogen. These negative effects were much more pronounced in cv. Messire compared to Protecta. These findings show the broad metabolic dampening effects of *D. pinodes* on *P. sativum* seeds, particularly for cv. Messire especially in the absence of AMF. Protecta, in contrast, shows a stronger increase in several proteins of the signaling, stress response, development, amino acid, glycolysis, and of the secondary metabolism, which might be related to the enhanced protection of cv. Protecta against the pathogen. Here, several of our previously identified metabolites and proteins involved in general pathogen resistance can be confirmed (Ranjbar Sistani et al., 2017).

For instance, we previously found proteins of the Late Embryo Abundant (LEA)-related family among the most significantly involved in pathogen resistance response (Ranjbar Sistani et al., 2017). Several groups of those (frv2_129189; frv2_25524, frv2_11113; frv2_54342, frv2_79176, P28639, frv2_128555) again showed a significant increase upon pathogen attack, however, independent of M treatment. LEA proteins are mainly known to be induced during seed development and in resistance to dehydration (Battaglia and Covarrubias, 2013). Our data confirm the findings of our previous work that LEA proteins are important for enhanced seed pathogen resistance and are not only involved in drought stress tolerance (Ranjbar Sistani et al., 2017).

Likewise, vicilin storage proteins (frv2_74601 and frv2_80935) were again found with a stronger increase under infection in cv. Protecta, however, slightly but not significantly stronger in M vs. NM treated plants. Nevertheless, this effect was more pronounced in rhizobia symbiotic plants (Ranjbar Sistani et al., 2017).

Also, the phytohormone abscisic acid (ABA) is a known component in plant environmental stress reactions (Luo et al., 2009; Song et al., 2014; as cited in Porcel et al., 2012; Adolfsson et al., 2017). A higher ABA content has been detected in leaves of *Medicago truncatula* colonized by AMF (Adolfsson et al., 2017). In Arabidopsis seeds, it has been reported that salicylic acid application induced ABA signaling leading to increased synthesis of ABA-regulated proteins, such as LEA proteins, dehydrins, and heat shock proteins, (Rajjou et al., 2006). Although here, ABA was notably enhanced in seeds upon pathogen infection, this accumulation was not enhanced in AMF symbiotic plants. Thus, our data support a role of ABA during pathogen defense and enhanced LEA protein levels independent on AMF symbiosis.

Interestingly, we found several plastidic proteins in pea seeds strongly depleted in both cultivars upon pathogen defense, independent of AMF symbiosis. Plastids are important components for embryos development in *P. sativum* (Smith et al., 1990). The role of chloroplast in the seeds biotic stress response, however, has not been described before. We suggest that the huge breakdown of plastidic proteins, we observed upon infection, might serve as a source for the enhanced secondary metabolite synthesis. The findings are in line with Fondevilla et al. (2011) who found that genes assigned to the primary metabolism were mostly down-regulated in accession P665, a resistant genotype, compared to Messire as susceptible pea cultivar against *D. pinodes* infection. Altogether, a common pathogen response of both cultivars is evident through enhanced levels of proteins involved in (iso) flavonoid production, of which the most significant and common compound identified was Sativan. Unfortunately, not much is known about the function of this isoflavone. Our data, however, support that it is involved in pathogen defense, especially of NM treated plants.

We found a significant reduction in seed infection levels, as well as lower levels of leaf lesion areas, when comparing M vs. NM treated plants of both cultivars. Remarkably, with an almost 2-fold reduction of seed infection levels the bio-control potential of mycorrhizal symbiosis against *D. pinodes* was very similar to the potential of rhizobial symbiosis demonstrated in our previous study (Ranjbar Sistani et al., 2017). This is particularly interesting because our earlier studies on AMF symbiosis showed no reduction in leaflets disease severity upon *D. pinodes* attack (Desalegn et al., 2016; Turetschek et al., 2017). Nevertheless, it has been reported that AMF protect plants against pathogens through the dampening of phytopathogenic effects (Borowicz, 2001). Also, AMF inhibiting impact on disease caused by *Xanthomonas translucens* on leaves of wheat was observed (Fiorilli et al., 2018). Altogether, the significant decrease of seed infection besides smaller lesion areas in AMF treated plants highlights the bio-control potential of AMF as a belowground microsymbiont reducing disease severity caused by an aboveground pathogen, in line with the previously reviewed findings by Jung et al. (2012), reporting the role of AMF in the reduction of infections caused by *Botrytis cinerea* (Møller et al., 2009; Pozo et al., 2010), *Alternaria solani* (Fritz et al., 2006; de la Noval et al., 2007), and *Magnaporthe grisea* (Campos-Soriano et al., 2012). Additionally, the high potential of AMF to control *B. cinerea* in tomato has been identified (Fiorilli et al., 2011). Nevertheless, compared to *Rhizobium* inoculation (Desalegn et al., 2016), AMF seems to have less significant effects on pea cultivar growth promotion and leaf protection but a similar impact on seed pathogen resistance. This study together with our earlier work (Ranjbar Sistani et al., 2017) revealed that both mycorrhizal and rhizobial below-ground symbionts are dominant against above-ground pathogen invasion. Besides, the below-ground symbiotic activity by AMF or *Rhizobium* reduced the systematic infection caused by the above-ground pathogen. Both studies indicate that the efficiency of these fungal and bacterial microsymbionts could be influenced by cultivar type and pathogen infection, however, their general bio-control function and infection inhibition is cultivar independent.

Levels of Vignatic acid A were increased in seeds of M treatments of cv. Messire upon pathogen infection. Vignatic acid A is known as a preventing compound with a promoting impact on resistance against bruchid beetle pest (Sugawara et al., 1996).

This cultivar specific effect becomes even more complex when comparing metabolic pathways that are induced by AMF symbiosis of healthy plants and are potentially involved in pathogen response. This specific mechanism (priming) of the plant metabolism by microsymbionts such as AMF, before infection by biotrophic and hemibiotrophic pathogens, is known as ISR (Cameron et al., 2013). Primed plants have already developed a certain metabolic defense apparatus, which reduces or even prevents pathogen attack. For instance, phytohormone JA plays a role in inducing systemic resistance against stresses (Luo et al., 2009; Song et al., 2014 as cited in Staudinger et al., 2016; Adolfsson et al., 2017; Bernardo et al., 2017) and also during plant development (Bernardo et al., 2017). Also, JA has a high impact on mycorrhizal colonization especially in higher plants (Gutjahr et al., 2015, as cited in Bernardo et al., 2017). According to our results, a protein related to the synthesis of JA (lipoxygenase frv2_95615 and frv2_84258) was highly increased in M plants (**Table S4**). Furthermore, data indicate that the JA-responsive protein (frv2_84258) is involved in ISR (**Figure 4** and **Table S6**). Additionally, we identified N-jasmonoyl isoleucine, and it was significantly enhanced in cv. Protecta, similar to the levels of JA in our previous study (Ranjbar Sistani et al., 2017), but this time strongly induced by AMF instead of *Rhizobium* symbiosis. The JA has a role in regulating of pea reaction against *D. pinodes* (Fondevilla et al., 2011). These findings support that cv. Protecta is not only a more resistant genotype but is also more receptive to ISR mechanisms by AMF. Hence, our data support that the effect of AMF treatment on the seed metabolism and in line with our previous findings of *Rhizobium* treatments is not due to a difference in P-regime but rather depending on the cultivar and due to a global change in metabolic homeostasis of the whole plant induced by microsymbiosis. Although pathogenesis-related (PR) proteins are known to be commonly involved in pathogen defense (van Loon et al., 2006), we found only a few of them involved in pea leaf and seed protection (Desalegn et al., 2016; Ranjbar Sistani et al., 2017). Here again, we found possible isoforms (frv2_110573; frv2_74661; frv2_41448; frv2_88778) induced upon pathogen infection in cv. Protecta seeds only. Interestingly, they were already induced by AMF symbiosis and further accumulated upon stress. Besides PR proteins, the detection of proteins involved in cell wall fortification, such as proteins of the monolignol pathway, seemed limited. Nevertheless, we found a UDP-glucosyltransferase as well as UDP-glucose 4-epimerase and UDP-glucose 6-dehydrogenase possibly involved in cell wall synthesis. These three enzymes were induced upon pathogen attack of cv. Protecta, but only UDP-glucosyltransferase (frv2_103598) was also enhanced in seeds of AMF symbiotic plants (cv. Protecta). Furthermore, Cyanidin 3-sophoroside 5-glucoside seemed involved in better seed pathogen resistance upon AMF symbiosis of cv. Protecta, being only induced upon infection of M treated plants. These findings suggest that AMF does not only prime.

The 2,3-Dihydroxy-2,4-cyclopentadien-1-one was also accumulated upon pathogen infection in both cultivars with a more significant effect on cv. Protecta. Very striking were also the high levels of several proteins of amino acid metabolism and glycolysis in cv. Protecta. Protein levels showed a strong increase under pathogen infection of non-mycorrhizal cv. Protecta, but some were also induced in healthy M treated plants and did not much further increase in Protecta seeds of M plants after pathogen attack, indicative for a primed primary metabolism as previously described by Schwachtje et al. (2018). By the production of pyruvate, the entry metabolite of the TCA cycle, glycolysis plays a role in energy supply and thus the production of defense metabolites (Rojas et al., 2014). Hence, the data suggest that the cultivar related difference in the primary metabolism seems another reason for the better performance of cv. Protecta.

Nevertheless, some defense mechanisms were also found to be more pronounced in cv. Messire. For instance, flavonoid Quercetin 3-(6''-acetylgalactoside)-7-rhamnoside strongly accumulated upon pathogen infection, especially in cv. Messire and most strongly in seeds of M treated plants. Similarly, Pisatin as specific phytoalexin of *P. sativum* has been induced against pathogen attack (De Wit-Elshove, 1969). Its synthesis pathway has previously been observed to be induced in the leaf proteome of *Rhizobium* inoculated pea plants under infection caused by *D. pinodes* (Desalegn et al., 2016). In contrast, pisatin involvement in rhizobial induced seed protection could not be detected in our previous study (Ranjbar Sistani et al., 2017). Interestingly, in the present study, a considerable AMF induced enhancement of proteins and metabolites of the secondary metabolism such as proteins of the flavonoid biosynthesis, especially the accumulation of isoflavone reductase and chalcone isomerase, along with increased levels of flavonoids and isoflavonoids, mainly in seeds of cv. Messire under disease stress was found. This finding supports that pisatin biosynthesis is induced in seeds of M plants upon pathogen infection.

**Figure 5:**
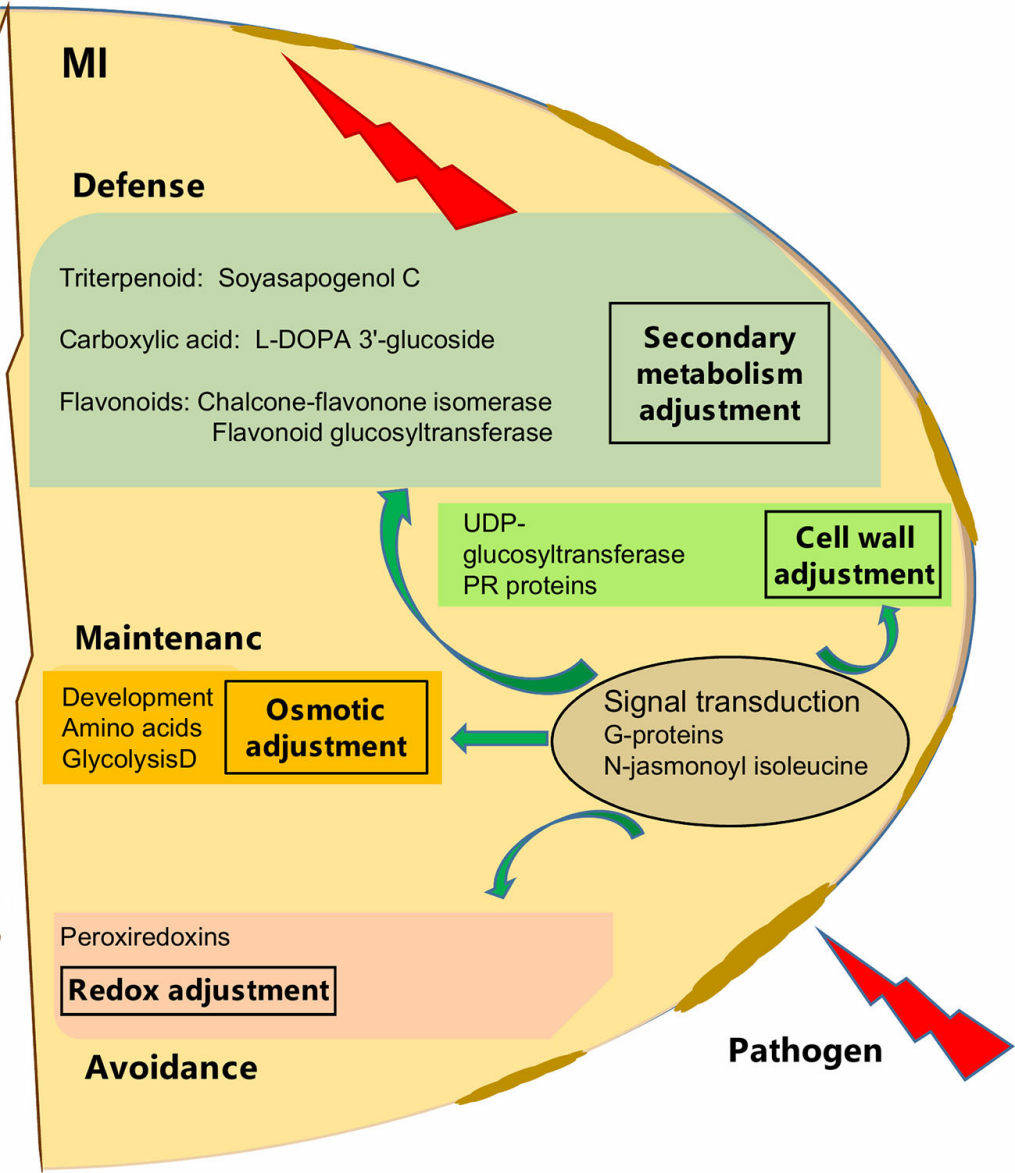
Schematic overview of mycorrizal induced response of pea seed (grown in pots) against *D. pinodes* infection. Seed metabolites and proteins significantly (Tukey HSD, p < 0.05; n = 3; **Supplementary Table S3B** and **S4**) enhanced with the highest ICA loadings (> 0.01 and < −0.01; **Supplementary Table S5**) are shown. MI, Mycorrhizae inoculated, and pathogen-infected.

Although, AMF showed cultivar specific effects, particularly for cv. Protecta, our results also indicated a general enhancement of pathogen resistance through AMF symbiosis (**Figure 5**). For example, G-proteins, known as signal transduction regulators (Smith et al., 1998; van Heusden, 2009; Smith et al., 2011; Parua and Young, 2014, as cited in Sun et al., 2018) have a role in plant responses to various stress conditions (Roberts et al., 2002; Lozano-Duran and Robatzek, 2015; Li et al., 2016, as cited in Sun et al., 2018). Mycorrhizal induced accumulation of G-proteins in wheat roots against drought stress was also reported (Bernardo et al., 2017). In our study, the group of proteins (frv2_86036; frv2_118099; Q9C5W6; frv2_94136) was particularly increased in seeds of AMF-associated plants under pathogen disease, indicating a possible role in ISR signaling.

In general, we found a similar molecular defense pattern induced by either AMF like *Rhizobium* against *D. pinodes*, described in our prior study (Ranjbar Sistani et al., 2017). This is not surprising, as the host builds fortification and munitions. Hence, Flavonoids and Soysapogenol C were important components in secondary metabolism adjustment in both *Rhizobium*- and AMF-induced responses. Interestingly, however, L-DOPA 3'-glucoside, discussed above, was significantly induced by AMF and in response to stress and not found responsive to *Rhizobium*-symbiosis. Also, the main proteins involved in signal transduction, mentioned above, were differentially induced. While, AMF enhanced levels of G-proteins and phosphatase 2C seems *Rhizobium* specific (Ranjbar Sistani et al., 2017). The analysis of secondary metabolites is still in its infancy and identification remains a bottleneck. Thus, major symbiont and pathogen involved differences and further key secondary metabolites regulation pathogen defense, remain to be identified in the future. The difference might also lie in response rate and/or induction level that might be different not only between cultivars but also depending on symbiosis, terms that need further evaluation.

Taken together, among common stress responses, several proteins and metabolites have been found AMF and cultivar specifically regulated, demonstrating different defense strategies. While genotype-specific resistance pattern is a common base in plant breeding, not much is known about genotype-specific microsymbiont induced priming effects.

## Conclusions

In this study, we demonstrated that AMF is not only promoting growth and yield of pea plants but also protects seed yield upon pathogen attack. The seed metabolic response of two cultivars with varying susceptibility to the pathogen *D. pinodes* was analyzed. Besides, with the growth-promoting effect of AMF microsymbiont, we demonstrate its positive impacts on the resistance of the plants against the pathogen. We found that the genotypic effects are strong for both the general and the AMF induced pathogen response.

Altogether, cv. Protecta, the less susceptible genotype, accumulates more proteins of the secondary metabolism involved in lipid, flavonoid, and phytohormone production as well as of the primary amino acid and glycolysis metabolism. Besides, cv. Protecta showed a stronger induction (priming) of seed metabolic pathways upon AMF symbiosis, which led to a dampened and less severe but more effective response to pathogen attack. Nevertheless, pathogen infection was also reduced in cv. Messire when interacting with AMF. However, infection levels were similar compared to cv. Protecta without AMF symbiosis and thus AMF impact less effective. Thus, the data demonstrate AMF and genotype-specific pathogen defense strategies. This study demonstrates that AMF as below-ground microsymbiont not only promote the growth above-below ground parts of pea plants but enhance the resistance of *P. sativum* plants and hence protects seed quantity. Also, AMF have a notable influence on the seed metabolome and proteome, influencing seed metabolism and thus nutritional and medicinal pea seeds quality.

Although, mycorrhizal and rhizobial symbionts in singular applications could be prescribed for improvement of pea seed protection, little is known about impacts of co-inoculation, often occurring in nature, and therefore investigations are demanded.

We believe that sustainable development of food resources for food poverty elimination can be strongly supported through the enhancement of productivity and quality of crops by using microbial symbiotic potentials. Focusing on plant species supplying food and microsymbiont interaction studies on improving production- and seed quality-related strategies under stress conditions besides promoting current ecological properties should be a priority in plant and agricultural studies. We recommend more studies related to efficiency assessment of soil microorganisms on food plants productivity upon abiotic and biotic stresses under field conditions with considering the environmental viabilities.

## Data Availability Statement

The datasets generated for this study can be found in the PRIDE PXD006617.

## Author Contributions

Experiments, including phenotyping, proteomics, and secondary metabolites analyses were carried out by NRS. Data mining was performed by NRS and SW. NRS and SW wrote the manuscript. SW, GD, and H-PK conceived the work. The manuscript was revised by GD and H-PK.

## Funding

We appreciated the support from COST Action FA1306.

## Conflict of Interest

The authors declare that the research was conducted in the absence of any commercial or financial relationships that could be construed as a potential conflict of interest.

## Footnotes

1 http://www.fao.org/faostat/en/#data/QC

2 https://invam.wvu.edu/methods/mycorrhizae/mycorrhizalength

3 www.massmatrix.org

4 http://www.hmdb.ca/

5 https://metlin.scripps.edu/

6 http://kanaya.naist.jp/KNApSAcK_Family/

7 http://www.statgraphics.com/

8 https://shiny.rstudio.com/

9 http://qvalue.princeton.edu/

## Supplementary Material

Figure S1 Comparison of functional categories (including > 2 proteins) of seed proteome significantly (Kruskal-Wallis; ANOVA, Tukey HSD test; p < 0.05) increased (summed increased fold change ≥ 2) in M vs. NM, I vs. U and Me vs. Pr. Me: cv. Messire, Pr: cv. Protecta, M: mycorrhizal, NM: non-mycorrhizal, I: infected (diseased), U: uninfected (healthy).
