# Supplementary figures and images for: Seed Metabolism and Pathogen Resistance Enhancement in Pisum sativum During Colonization of Arbuscular Mycorrhizal Fungi: An Integrative Metabolomics-Proteomics Approach
Source: Front Plant Sci. 2020 Jun 12;11:872. doi: 10.3389/fpls.2020.00872 (PMC7309134; doi:10.3389/fpls.2020.00872)

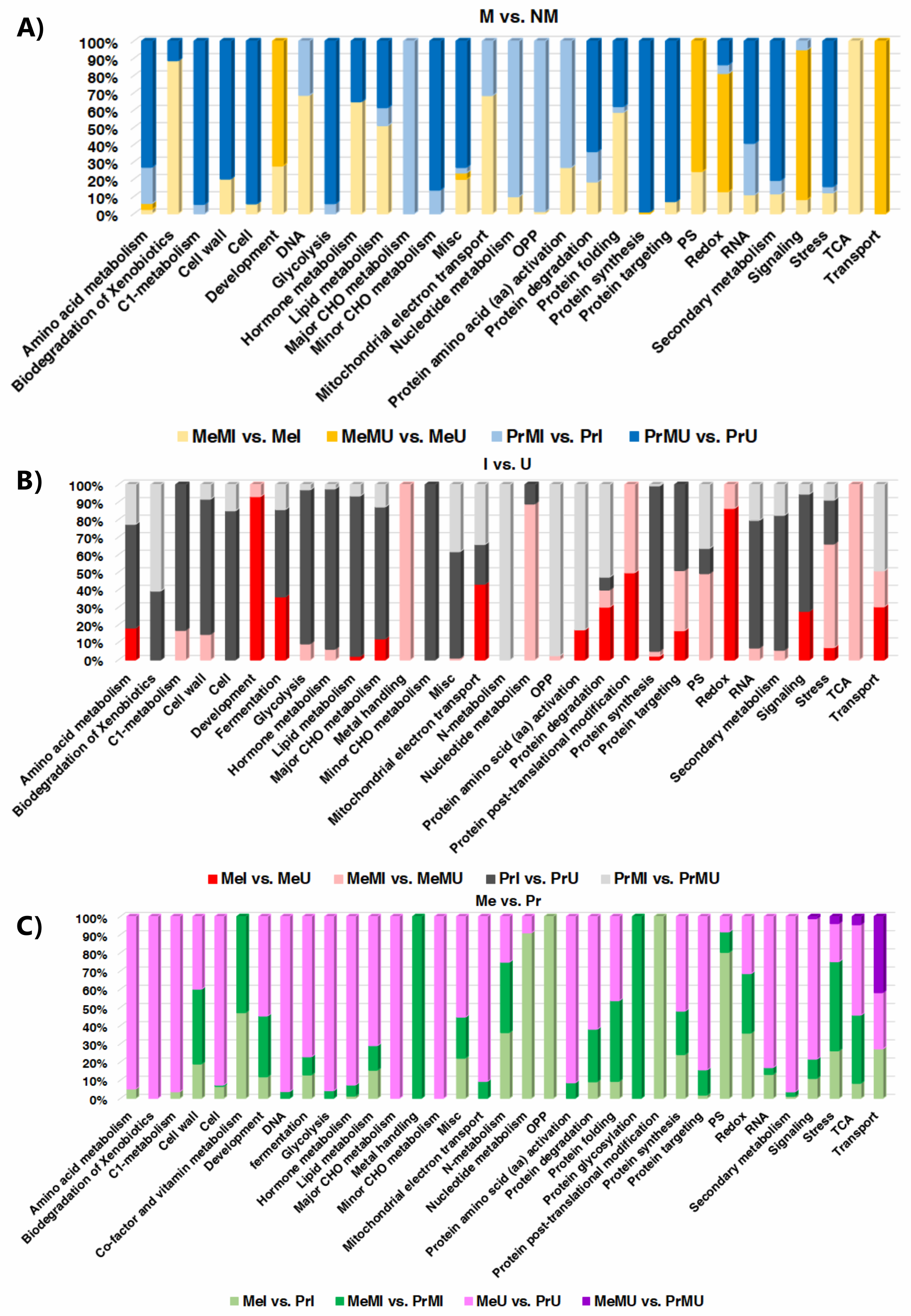

Supplement: Figure S1 — Comparison of functional categories (including > 2 proteins) of seed proteome significantly (Kruskal-Wallis; ANOVA, Tukey HSD test; p < 0.05) increased (summed increased fold change ≥ 2) in M vs. NM, I vs. U and Me vs. Pr. Me: cv. Messire, Pr: cv. Protecta, M: mycorrhizal, NM: non-mycorrhizal, I: infected (diseased), U: uninfected (healthy). [file Image_1.tif]
