# Supplementary material for: Seed Metabolism and Pathogen Resistance Enhancement in Pisum sativum During Colonization of Arbuscular Mycorrhizal Fungi: An Integrative Metabolomics-Proteomics Approach
Source: Front Plant Sci. 2020 Jun 12;11:872. doi: 10.3389/fpls.2020.00872 (PMC7309134; doi:10.3389/fpls.2020.00872)
Supplement: Supplementary file 2 [file DataSheet_1.zip › Sup Tables/Sup Table 1 and 2.docx]

Supplementary Material

Seed Metabolism and Pathogen Resistance Enhancement in *Pisum sativum* During Colonization of Arbuscular Mycorrhizal Fungi: An Integrative Metabolomics-Proteomics Approach

Nima Ranjbar Sistani, Getinet Desalegn, Hans-Peter Kaul and Stefanie Wienkoop*

*** Correspondence:** Dr. Stefanie Wienkoop: stefanie.wienkoop@univie.ac.at

**
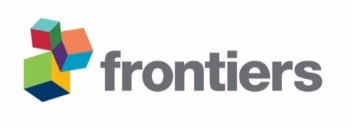
**

**Table S1** Mycorrhizal (AMF) root colonization, estimated total mycorrhizal root length (TMRL) and estimated total plant root length (TPRL) of *Pisum sativum*.

| **Treatments** | **AMF root colonization (%)** | **TMRL (cm)** | **TPRL (cm)** |
| --- | --- | --- | --- |
| **MeMI** | 64.78±5.09 a | 156.73±81.21 a | 173.56±80.85 a |
| **MeMU** | 67.84±5.09 a | 285.19±81.21 a | 300.27±80.85 a |
| **PrMI** | 67.29±5.09 a | 401.39±81.21 a | 418.64±80.85 a |
| **PrMU** | 80.13±5.09 a | 284.56±81.21 a | 292.64±80.85 a |

TMRL: total mycorrhizacolonized root length, TPRL: total plant root length, Me: cv. Messire, Pr: cv. Protecta, M: mycorrhizal, NM: non–mycorrhizal, I: infected (diseased), U: uninfected (healthy). Values represent the means ± standard error of twenty root samples per biological replicate (pot). Different letters per column indicate significant (Tukey HSD test, p < 0.05) differences among treatments.

**Table S2** The correlation coefficients (Pearson) upon paired analyses of mycorrhizal root colonization, TMRL, TPRL, seed number per plant, root biomass and non–soaker index (rang: -1, 1).

|  | **AMF root colonization (%)** | **TMRL (cm)** | **TPRL (cm)** |
| --- | --- | --- | --- |
| **TMRL (cm)** | 0.34 |  | 1.00 *** |
| **TPRL (cm)** | 0.31 | 1.00 *** |  |
| **Seed number per plant** | 0.78 ** | 0.32 | 0.30 |
| **Root–FW (g)** | 0.39 | 0.79 ** | 0.78 ** |
| **Root–DW (g)** | 0.38 | 0.84 *** | 0.83 *** |
| **Non–soaker index** | (-0.63) * | (-0.26) | (-0.25) |

TMRL: total mycorrhizal root length, TPRL: total plant root length, DW: dry weight and FW: fresh weight. Significant (* (p < 0.05), ** (p < 0.01) and *** (p < 0.001)).
